# STUDENT CARE PARTNERING AMONG STUDENTS AT A HBCU: EXPLORING THE POTENTIAL IMPACT ON STUDENT PERSISTENCE

**DOI:** 10.1093/geroni/igac059.3108

**Published:** 2022-12-20

**Authors:** Claudia Thorne

**Affiliations:** Coppin State University, Baltimore, Maryland, United States

## Abstract

The number of students enrolled in higher education who are taking care of older adults, many of whom may be suffering from an illness, is expanding. The numerous factors in care partnering can compromise student success and persistence towards graduation. While most care partners experience challenges, the care partnering experience of Black students is magnified because Historically Black Colleges and Universities (HBCUs) have a higher enrollment of nontraditional and first-generation college students. Students may be primary care partners who provide most of the care, or they may provide supportive care as secondary, tertiary, and auxiliary care partners within an extended family or kinship network. Aging in place, within the context of family and community, emerges from African culture and tradition deeply grounded in filial loyalty. Care partnering is nuanced in the interaction of race, ethnicity, gender, family relationships, and student status; however, little is known about the lived experience of Black student care partners and how to support them to facilitate their persistence towards graduation. This poster describes initial efforts to understand Black student experiences in care partnering in the context of a Mid-Atlantic HBCU and suggests the next steps in a multi-year research program.

